# Relative Pose Determination of Uncooperative Spacecraft Based on Circle Feature

**DOI:** 10.3390/s21248495

**Published:** 2021-12-20

**Authors:** Yue Liu, Shijie Zhang, Xiangtian Zhao

**Affiliations:** Research Center of Satellite Technology, Harbin Institute of Technology, Harbin 150080, China; y.liu@stu.hit.edu.cn (Y.L.); zhaoxiangtian@hit.edu.cn (X.Z.)

**Keywords:** on-orbit service, uncooperative spacecraft, relative pose, ToF camera, circle features

## Abstract

This paper investigates the problem of spacecraft relative navigation with respect to an unknown target during the close-proximity operations in the on-orbit service system. The serving spacecraft is equipped with a Time-of-Flight (ToF) camera for object recognition and feature detection. A fast and robust relative navigation strategy for acquisition is presented without any extra information about the target by using the natural circle features. The architecture of the proposed relative navigation strategy consists of three ingredients. First, a point cloud segmentation method based on the auxiliary gray image is developed for fast extraction of the circle feature point cloud of the target. Secondly, a new parameter fitting method of circle features is proposed including circle feature calculation by two different geometric models and results’ fusion. Finally, a specific definition of the coordinate frame system is introduced to solve the relative pose with respect to the uncooperative target. In order to validate the efficiency of the segmentation, an experimental test is conducted based on real-time image data acquired by the ToF camera. The total time consumption is saved by 94%. In addition, numerical simulations are carried out to evaluate the proposed navigation algorithm. It shows good robustness under the different levels of noises.

## 1. Introduction

The on-orbit service for failed spacecraft has been widely concerned and studied since the 1960s. In order to extend their lifetime in space, the USA, Europe, Japan, Canada, Russia, China, and other countries and regions presented many approaches for on-orbit service, such as replacement of device modules, fuel injection and on-orbit assembly and maintenance [1,2,3,4,5]. Most of these methods rely on the spacecraft rendezvous and docking (R&D) technology. High precision relative measurement is the prerequisite for R&D process, especially during the close-proximity operations.

According to the measured objects, the spacecraft relative measurement can be divided into cooperative and uncooperative types. Compared with relatively mature cooperative pose determination techniques [6], the relative measurement for uncooperative targets without special recognition devices is more challenging [7]. Electro-optical sensors used for uncooperative relative measurements include passive ones based on the visible band like monocular and stereo vision systems, and active sensors based on the infrared band like LIDAR [8]. Passive sensors are easily affected by light conditions, which is difficult to guarantee the image quality under dynamic, while active sensors can adapt to more application scenarios in space. “Time-of-Flight (ToF) camera” is a specific type of LIDAR, which uses either pulse or continuous wave to measure the time of flight based on detector arrays and can simultaneously providing intensity data and range information for every pixel at a high frame rate [9]. With low weight, low power consumption, compact structure, and robustness to illumination changes, many LIDAR companies are committed to the research on miniaturization, low cost and high performance of the camera and ToF camera gain a rapid development in the civil field, which receives extensive attention in the aerospace field [10,11,12].

For uncooperative targets, the general framework of pose determination is divided into two steps, acquisition and tracking. When dealing with the acquisition step, an initial pose is solved fast using the first dataset without a priori pose information [13]. This pose result allows for residuals, but too much error will lead to failure of tracking. In this paper, the acquisition process is investigated. Although the pose result is not accurate enough, a fast convergence can be made in the tracking step.

Feature-based methods are most commonly used in the process of acquisition [14,15,16]. Natural features on uncooperative targets are detected by sensors and the corresponding pose can be obtained by matching them with a benchmark such as a model [11,17]. Natural circle features such as docking ring and nozzle are common attributes of most spacecraft and are more stable compared with point and line features and are used as a reference feature in many missions [6,18,19]. In this paper, the pose estimation does not need the whole model or specific local model and the unique condition is the presence of circle features on the spacecraft. However, even considering the blurring problems [20], there are still shadows, overexposure and other problems in actual scenes and circle feature detection is often affected by the lighting conditions, the geometry of the circle feature and the shooting perspective, resulting in poor circle detection results of the images. Some scholars have given solutions from different views for the problems in different scenes [21,22,23].

**Figure 1:**
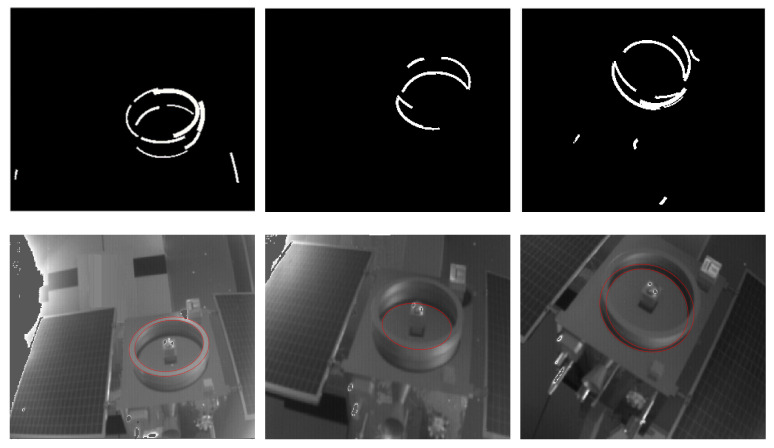
Different types of ellipse-detection results.

During the study of circle features in this paper, the circle images are easily shaded by the geometry of the circle features. In addition, in the tests of two ellipse detection algorithms [24,25], multiple independent circle feature structures are often confused as the same ellipse as shown in Figure 1 (i.e., an ellipse detection result contains multiple structures of the actual target). Other algorithms sometimes have the same questions [23]. If we rely only on ellipse detection algorithms for circle feature extraction, the results during the actual target motion often do not make the pose reliable.

**Figure 2:**
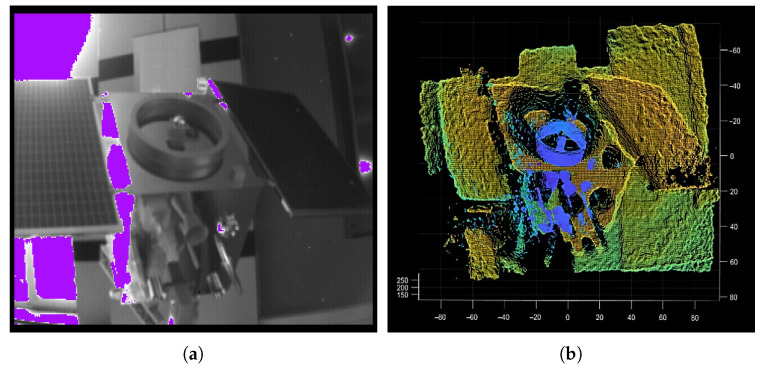
Data of Time-of-Flight (ToF) camera. (**a**) The captured gray image; (**b**) The captured point cloud in the field of view (76,800 points). Obviously, the circle feature only accounts for a small part of the point cloud.

For the segmentation of the point clouds, some specialized segmentation algorithms are studied from the complete model [26,27]. However, the point cloud data at a certain moment (in the acquisition process) is the result of a certain angle of view of the target spacecraft. Due to the structure occlusion, the point cloud will be incomplete, and the circle feature may also be incomplete, which will make circle feature segmentation more difficult than simple geometric features. Moreover, the circle feature is local, and the direct segmentation (or direct alignment) of the point cloud will result in a large scale of point cloud segmentation and low calculation efficiency [11] (as shown in Figure 2).

Therefore, image-assisted point cloud segmentation methods are considered [28]. The gray image and depth image of the ToF camera share one optical imaging system and have the same camera parameters. Gray and depth images are associated by pixels, one-to-one correspondence, which avoids the complex registration between two types of sensors and data. In addition, the ToF camera can keep constant accuracy in the range measurement as the target distance varies. In this paper, we will investigate the above problems using a ToF camera and propose a fast segmentation method using the gray image to assist point clouds according to the characteristic of the ToF camera and some tricks, which solve the problems of large segmentation scale and structure occlusion caused by direct segmentation of point clouds, as well as the problem of ellipse detection errors due to shadows and multiple circles in gray images.

After the circle features are extracted, this paper will divide the irregular circle features point cloud into two general groups to meet the different circle feature requirements in different scenes and fit the parameters. Finally, the relative pose can be solved by using the circle feature parameters. Because the relative pose solution model uses the circle features, therefore a special coordinate system is built to simplify the pose calculation. The paper conducts a large number of experiments to verify the effectiveness of the algorithm in each part. For the effect of circle feature extraction, we use actual shooting data for verification. For the effect of the whole framework of the positional solution, the numerical simulation will be used for the accuracy evaluation.

The paper is organized as follows: The architecture of the proposed method and basic calculation model is in Section 2. The main part of the relative pose determination algorithm is described in Section 3 and Section 4. Section 5 presents the experimental results. Finally, Section 6 concludes the paper.

## 2. Architecture of the Proposed Method

**Figure 3:**
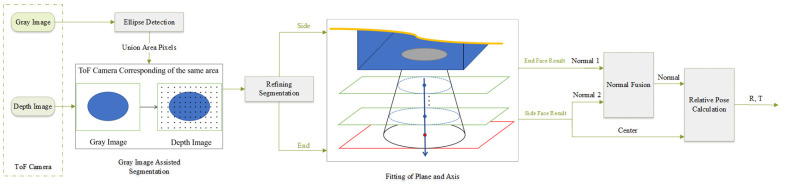
Illustration of coordinate frames and related vectors.

The ToF camera-based uncooperative target relative pose fusion calculation algorithm using circle features for on-orbit service mainly has three steps: (1) circle feature detection and segmentation, (2) parameter fitting for space circle, and (3) relative pose calculation by circle parameters. The architecture of the algorithm is shown in Figure 3. It takes the gray image and the depth image of the ToF camera as input. At first, gray image auxiliary segmentation as fast initial segmentation is executed, which includes ellipse detection and point cloud transformation. It aims at reducing the actual processing scale of the point cloud and improve the efficiency of the algorithm. After segmentation, the local point cloud including circle features can be obtained. According to the data characteristics, the method calculates the circle parameters respectively and then fuses the results into a more robust one. Finally, the relative position and Euler angle (or transformation matrix) are calculated by the circle parameters according to the model in Section 2.2.

### 2.1. Coordinate Systems

**Figure 4:**
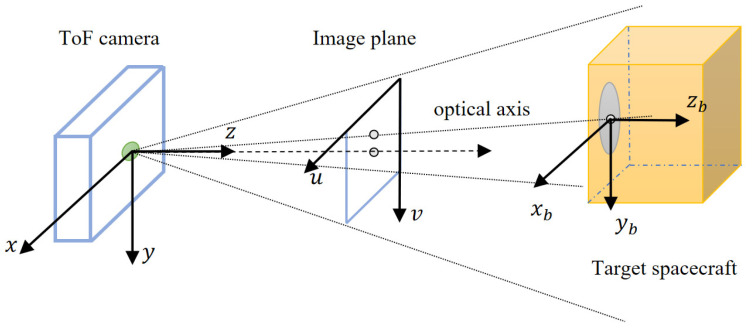
Definition of the coordinate system.

In this paper, the relative pose of the target spacecraft with a circle feature on the surface is measured by using the depth image and gray image of the ToF camera. To facilitate calculation and conversion, the following coordinate system is defined as follows (Figure 4):

The service spacecraft body frame (SBF) is the classical principal axis of the inertia coordinate system. It should be noted that the aimed relative pose of the uncooperative spacecraft is in the SBF.

The ToF camera reference frame (CRF) is the ToF camera coordinate system whose *z*-axis is directed along the optical axis.

Circle feature frame (CFF) is coincident and aligned with the target body frame (TBF), which can simplify the process of coordinate system transformation. The CFF’s *x*–*y* plane is located at the end face of the circle feature far from the mounting surface, and the origin is the center of the annular torus. The *z*-axis is perpendicular to the circle feature and points to the direction of the target spacecraft body.

The image frame (IF) consists of two parts. One is the gray-image coordinate system, and the other is the depth-image coordinate system. Both share the same optical system, so the image coordinate system is the same. IF takes the corner of the pixel above and left of the image as the origin.

Since the installation position of the ToF camera on the servicing spacecraft is known, the transformation relationship between the SBF and CRF is constant and can be calibrated in advance. Without loss of generality, the CRF is assumed to coincide with the SBF.

In this paper, the CFF is used as the TBF, and the centroid of the target spacecraft is not considered. The relative pose of the uncooperative target can be simplified as the transformation between the CFF and CRF. This result can be applied to the initial pose acquisition.

### 2.2. Relative Pose Calculation Model

For a circle feature, the parameters are the center, the radius, and the normal vector of the plane. This paper will use these circle feature parameters to study the spacecraft pose solution method.

Assume that the circle feature parameters are the center of the circle $r_{0}=\left(x_{0},y_{0},z_{0}\right)$, and the unit normal vector $n=(n_{x},n_{y},n_{z})$. These parameters are measured by the ToF camera in the CRF. Since the TBF is established on the circle feature, the displacement of the uncooperative target relative to the service spacecraft is the coordinate of the center of the circle in the CRF that is $T=r_{0}$.

For the unit normal vector of the circle feature, it is in the same direction as the *z*-axis of the TBF. After the vector is translated to the origin of the CRF, the corresponding rotation angle can be further obtained according to the geometric relationship, and then the rotation matrix of the uncooperative target relative to the service spacecraft is also obtained.

**Figure 5:**
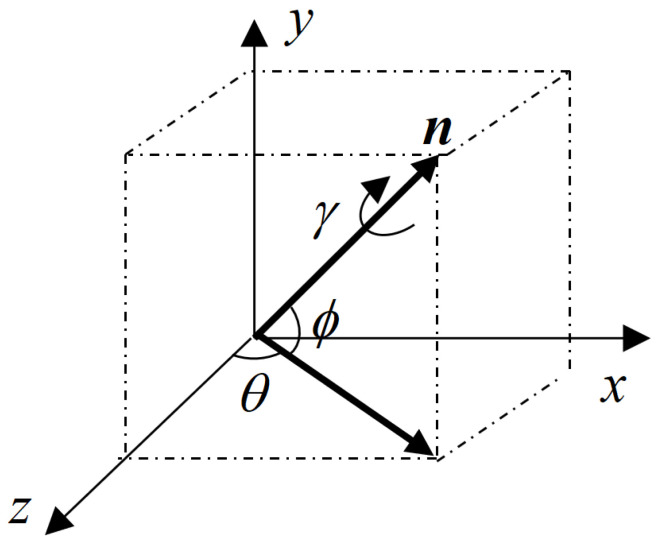
Schematic diagram of using the normal vector to solve the Euler angle.

Figure 5 shows a schematic diagram of the normal vector transferred to the CRF. For the convenience of calculation, the *z*-axis component of the unit normal vector obtained by the measurement is adjusted to a positive value, that is, the normal vector of the specified circle feature points to the body of the uncooperative target.

The geometric relationship can be obtained from Figure 5,

(1) $$\phi =\arctan\frac{n_{y}}{\sqrt{n_{x}^{2}+n_{z}^{2}}}$$

(2) $$\theta =\arctan\frac{n_{x}}{n_{z}}$$

where ϕ is the pitch angle and θ is the yaw angle.

The rotation matrix can be obtained as $R=C_{x}\left(-\phi \right)C_{y}\left(\theta \right)$: where

$$C_{x}=\left[\begin{array}{ccc} 1 & 0 & 0\\ 0 & \cos(-\phi ) & \sin(-\phi )\\ 0 & -\sin(-\phi ) & \cos(-\phi ) \end{array}\right],C_{y}=\left[\begin{array}{ccc} \cos\theta & 0 & -\sin\theta \\ 0 & 1 & 0\\ \sin\theta & 0 & \cos\theta \end{array}\right].$$

As a result, the use of circle features to calculate the position and attitude of the uncooperative spacecraft relative to the CRF can be realized. It should be noted that, due to the symmetry of the circle feature, the angle of rotation around the axis of symmetry cannot be calculated, which is not taken into account in this article. It is assumed that there is no rotation around the axis of symmetry of the circular feature.

### 2.3. ToF Camera Measurement Model and Data Transformation

**Figure 6:**
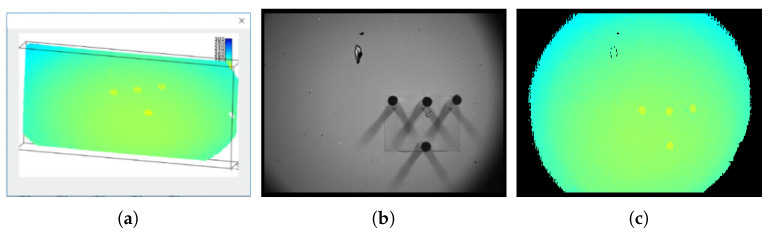
Data of ToF camera. (**a**) point cloud (76,800 points); (**b**) gray image; (**c**) depth image.

The sensor used in this paper is the ToF camera. The ToF camera integrates multiple functions and can collect multiple types of data, including the target distance, the intensity of the reflected laser, the spatial three-dimensional coordinates, and the target surface amplitude intensity (some types of the data are shown in Figure 6). The most typical and common data used are gray images and depth images or point clouds.

In particular, the depth image records the distance of the reflection point. If needed, the depth image can be converted into a point cloud by the camera internal parameters.

Assume that the ToF camera parameters are as follows: the focal length per pixel is $\left(f_{x},f_{y}\right)$ and the principal point is at $\left(u_{c},v_{c}\right)$.

Before calculating, the origin of the image coordinates is needed to translate to the position of the principal point, and then the pixel coordinates on the translated depth image are $\left(u_{r},v_{r}\right)=\left(u,v\right)-\left(u_{c},v_{c}\right)$.

The relationship between the pixel coordinates and the actual space coordinates of the target can be obtained from the principle of camera imaging: $\frac{v_{r}}{f_{x}}=\frac{x_{0}}{z_{0}}$, $\frac{u_{r}}{f_{y}}=\frac{y_{0}}{z_{0}}$.

According to the Time of Flight imaging principle of the ToF camera, the depth value on the pixel is the distance from the emitter to the reflection point. Thus, the constraint $x_{0}^{2}+y_{0}^{2}+z_{0}^{2}=d^{2}$ exists.

The space coordinate $\left(x_{0},y_{0},z_{0}\right)$ of the target reflection point corresponding to the pixel point is as follows:

(3) $$\begin{array}{cc} z_{0} & =\frac{d}{\sqrt{1+\frac{v_{r}^{2}}{f_{x}^{2}}+\frac{u_{r}^{2}}{f_{y}^{2}}}}\\ x_{0} & =\frac{v_{r}}{f_{x}}z_{0}\\ y_{0} & =\frac{u_{r}}{f_{y}}z_{0} \end{array}$$

## 3. Initial Point Cloud Segmentation Assisted by the Gray Image

**Figure 7:**
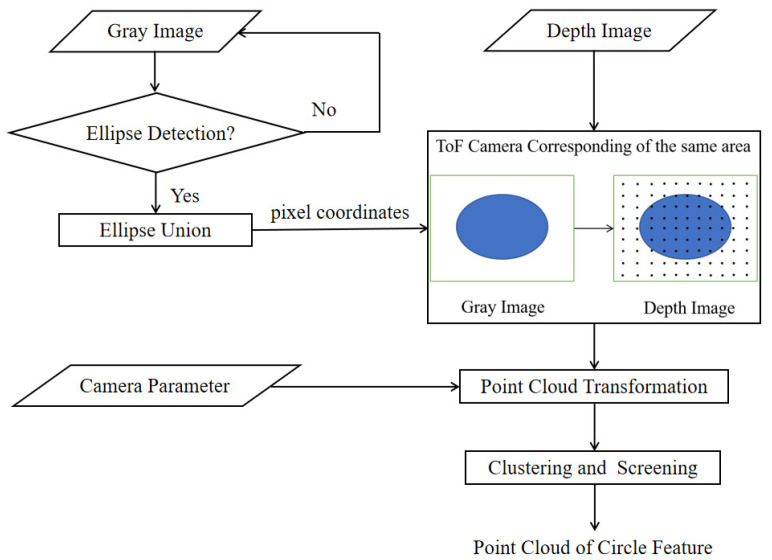
The flowchart of circle feature point cloud extraction.

Since the point cloud of the ToF camera containing depth information is the main basis for the subsequent parameter solving, the first step of calculating the relative pose of the uncooperative target is to extract the circle feature point cloud. However, the circle features in the actual scene may lead to poor circle feature extraction (as described in Section 1). The following will take advantage of the characteristics of the ToF camera and use the gray image to assist the point cloud segmentation, and employ some tricks to extract the circle feature point cloud even when the ellipse detection results are poor (the process is shown in Figure 7).

Firstly, an ellipse detection algorithm is used in the gray image, and the results are taken to form a union, which ensures that the region contains as many circle feature structures as possible. The ToF camera parameters are used to transform the depth image to a small-scale point cloud obtained from the corresponding region containing a circle feature structure. After further simple denoising and segmentation, the point cloud of the circle feature area can be obtained.

As a basic technology in image processing, ellipse detection is widely used in many practical problems. However, due to the complexity of the ellipse parameters, the image may suffer from motion blur, occlusion, unstable lighting conditions, noise, etc. in the motion scene, the ellipse detection algorithm still faces the challenge of efficiency, accuracy, and stability.

**Figure 8:**
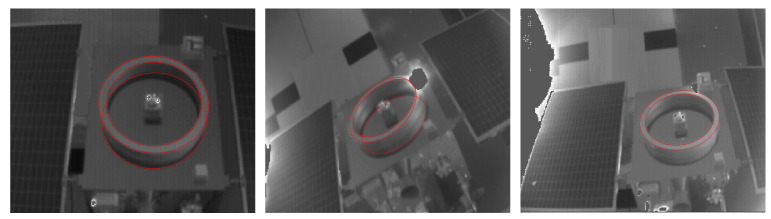
Some good results of ellipse-detection based on arc support.

When selecting an ellipse detection algorithm, in addition to the effect and stability, the form of the detection result must also be considered. The current ellipse detection algorithm results mainly have two types. One is the ellipse arc with width [25], and the other is the complete edge of the ellipse (Figure 8). This paper finally chooses an efficient and fast ellipse detection algorithm based on arc support proposed by Changsheng Lu in [24]. The result is a complete ellipse with more information, and the result is relatively stable.

**Figure 9:**
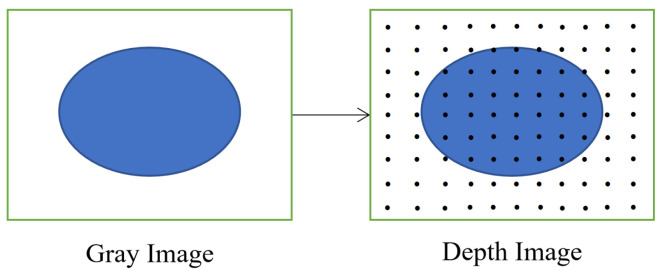
Gray image and depth image correspond to the same area.

When the ellipse detection is achieved, the parameters and edge pixel coordinates of the ellipse can be obtained, and the area containing the edge pixel is the area where the circle feature is located. Since the pixel position of the depth image in the ToF camera corresponds to that of the gray image one-to-one, the area containing the circle feature is also in the same area in the depth image (Figure 9). Using the ToF camera parameters to convert the depth image coordinates of the area into a point cloud, a small-scale point cloud containing a circle feature area can be obtained.

In the actual detection result, due to the width and height of the ring, shadows may be generated under certain lighting conditions, and arcs from different surfaces may be combined into the same ellipse, which may result in multiple ellipses. To obtain more effective information, the detected ellipse area is merged, and the point cloud converted from the depth image in the corresponding area will contain more complete circle features.

The circle feature point cloud extracted by the result of gray image ellipse detection contains not only the circle feature but also other interferences, such as the installation surface of the circle feature, and the false detection of the circle area because of the shadow area of a small part of the ring.

For the installation surface, it can be removed by plane segmentation easily.

The remaining part is segmented using clustering based on Euclidean distance to separate the group containing circle features. Suppose the segmented point cloud is $P_{\mathrm{seg}}$, and the total number of contained points is $N_{\mathrm{seg}}$. Searching the set of neighboring points by the minimum Euclidean distance $d_{min}$ with each internal point $p_{i}\left(1\leq i\leq N_{seg}\right)$ as the center, if the internal point already contains the label *L*, then skip it and search the next point. If the internal point has no label and the neighbor points also have no labels, a new label is assigned to the points in the area. If the internal point has no label and the neighbor points have labels, the smallest label in the neighbor is assigned to all points. After the clustering process, a total of $N_{L}$ labels are generated, and each point has its label.

The points containing the same label $\left\{p_{1}^{k},p_{2}^{k},\cdots ,p_{n_{k}}^{k}\right\}\left(1\leq k\leq N_{L}\right)$ are extracted to form a new point cloud $P_{k}$, containing $n_{k}$ points. Then, we have $P_{seg}={⋃}_{N_{L}}P_{k}$, $N_{seg}=\sum_{N_{L}}n_{k}$. The new point clouds are sorted by descending order according to the number, and the number difference of adjacent point clouds is calculated. When the number difference exceeds the threshold $n_{0}$, all the point clouds before this point cloud are considered as the point cloud of the final circle feature $P_{\mathrm{circle}\;}={⋃}_{m}P_{k}\left(1\leq m\leq N_{L},n_{m+1}-n_{m}>n_{0}\right)$.

Suppose the parameter not related to the vector in the plane obtained by fitting is $d_{f}$ (setting the *z*-axis component of the vector is positive). For the interference of a small amount of point cloud such as the shadow area, point cloud denoising can directly remove these discrete points. Thus, the point cloud segmentation aided by gray image is completed.

## 4. Feature Parameters Calculation of the Non-Ideal Circle

Some studies on the fitting of circle features have focused on the more regular geometry of the circle features [29,30], while the actual circle features may contain multiple structures or more complex structures such as docking ring, nozzle, etc. To make full use of the data and adapt to the circle features of more structures, this paper divides the circle features into the following two groups—the end surface away from the mounting surface and the side surface. The end surface is a flat structure, and the side surface is mainly a curved surface formed by rotating around an axis:

(4) $$P_{\mathrm{circle}\;}=P_{\mathrm{end}\;}\cup P_{\mathrm{side}\;}$$

### 4.1. Circle Feature Parameters Calculation of Each Group of Point Cloud

For the end face part, since it is only a plane structure, only the plane normal vector fitting is needed to obtain the normal vector of the target circle feature. Suppose the fitted plane is $ax+by+cz+d_{0}=0$, and the normal vector is $n_{\mathrm{end}}=\left(a,b,c\right)$. The radius *r* and center of the circle feature $r_{0}^{\mathrm{end}}$ can be estimated by the circle fitting of the points on the end surface and then the displacement is $t_{\mathrm{end}\;}=r_{0}^{\mathrm{end}\;}$.

**Figure 10:**
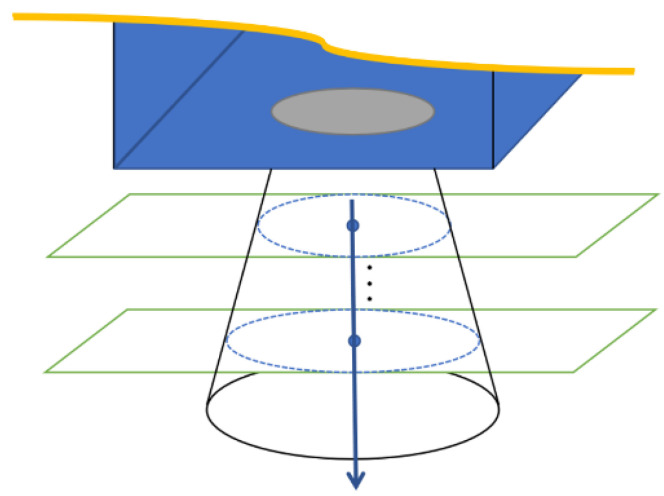
Axis fitting by the intersecting line.

For the side curved surface, the normal vector of the target circle feature is its axis. To adapt to more rotating body structures, the method of geometric cross-section fitting is adopted, using several planes parallel to the end face to intercept the side surface, and the obtained intercepting circle is fitted to the center. The axis is the straight line where the center of the circle is located (as shown in Figure 10).

Set the plane where the end face is located as the standard plane, which is $\Omega_{0}:ax+by+cz+d_{0}=0$. For the plane family parallel to the standard plane on the side surface, the function is $\Omega :ax+by+cz+d=0\left(d_{0}\leq d\leq d_{f}\right)$, where the plane parameter of the installation surface is $d_{f}$. Suppose the parameter of the selected section is $d_{j}$, and take *n* random numbers between $d_{0}$–$d_{f}$ to get the set of *n* plane dots. Then, fit the center of the circle, and fit the centers to get the normal vector direction $n_{\mathrm{rot}}$:

(5) $$d_{j}=\mathrm{rand}\left(d_{0},d_{f}\right)\left(j\in \left[1,n\right]\right)$$

(6) $$\Gamma_{j}=\left\{\left(x_{i},y_{i},z_{i}\right),ax_{i}+by_{i}+cz_{i}+d_{j}<\varepsilon \right\}$$

(7) $$O_{j}=\;\mathrm{fit}{\;}_{-}\mathrm{center}\;\left(\Gamma_{j}\right)$$

(8) $$L=\;\mathrm{fit}{\;}_{-}\mathrm{line}\left(O_{j}\right)=n_{\mathrm{rol}\;}t+p\left(p\in O_{j},t\in R\right)$$

Then, the normal vector of the space circle is $n_{\mathrm{side}}=n_{\mathrm{rot}}$.

For the center of the target circle feature, it is the center of the end face, that is, the intersection point of the side surface axis and the end face. After finding the rotation axis of the side surface, the center of the circle can be obtained by using the axis equation and the end face equation, which is $r_{0}^{\mathrm{side}\;}=L\cap \Omega_{0}$. Then, the displacement obtained in this part is $t_{\mathrm{side}\;}=r_{0}^{\mathrm{side}\;}$.

### 4.2. Parameters Fusion Based on Point Cloud

After the circle feature parameters calculation, both the normal vector and circle center get two results. To obtain the unique circle feature parameters to determine the relative pose of the uncooperative spacecraft, it is necessary to perform information fusion.

Performing a weighted summation of different results is the simplest and most effective way. For the parameter calculation method, the circle feature is divided into two groups which are the end surface and the side surface, and the result is solved in different ways. Therefore, the weight of each component of the normal vector or displacement is the ratio of the number of the divided point cloud and the total number *N* of feature point clouds before segmentation. The parameter weight of the end surface and the side of the circle feature point is

(9) $$w_{\mathrm{end}\;}=\frac{n_{\mathrm{end}\;}}{N},w_{\mathrm{side}\;}=\frac{n_{\mathrm{side}\;}}{N}$$

Then, the result of the normal vector and displacement of the circle feature is

(10) $$n=w_{\mathrm{end}\;}n_{\mathrm{end}\;}+w_{\mathrm{side}\;}n_{\mathrm{side}\;}$$

(11) $$t=w_{\mathrm{end}\;}t_{\mathrm{end}\;}+w_{\mathrm{side}\;}t_{\mathrm{side}\;}$$

Then, according to the relationship between the normal vector of the circle feature parameter and the rotation matrix, the rotation matrix R can be solved.

## 5. Experiments and Numerical Simulations

### 5.1. Circle Feature Point Cloud Segmentation Experiment

This part tests the effect of point cloud segmentation assisted by the gray image in the ToF camera. The data including gray images and depth images are obtained by shooting the satellite model with the circle feature using the ToF camera. The size of the images is 320 × 240 pixels. After calibration, the result is that the pixel position of the main point is [165.39, 122.44], the focal length is [394.16, 393.89], and the distortion coefficient is [−0.5953, 1.2452]. For the convenience of representation, the unit of the converted CRF is unified to centimeters. The computer host in this experiment is configured with an Intel i7 processor, 2.8 GHz of the main frequency, and 8 GB memory.

To test the improved efficiency of the segmentation method in this paper, the direct segmentation of the circle feature from the global point cloud is chosen for comparison. However, the circle feature is incomplete and contains multiple geometric structure disturbances, so it is difficult to directly use the circle/cylinder-based segmentation method [31]. Thus, the planar background segmentation and the clustering algorithm mentioned in Section 3 are used for the simple and effective method of separating the circle feature from the global point cloud.

**Figure 11:**
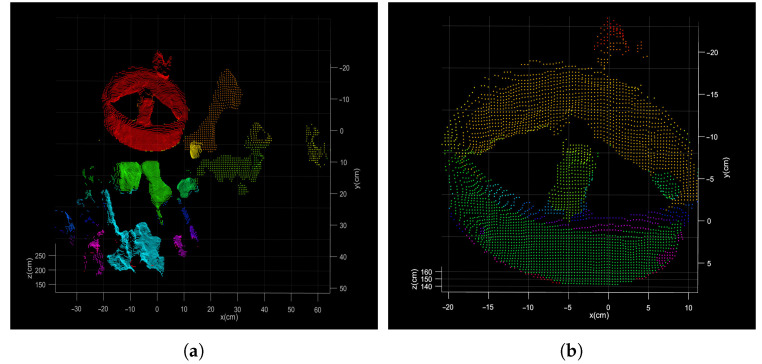
Direct segmentation result of point cloud. (**a**) the result after the planar background segmentation; (**b**) the result after the clustering segmentation.

As shown in Figure 2, there is a gray image and a point cloud obtained simultaneously by shooting. The point cloud (76,800 points) is obtained by converting the depth image with the camera parameters. After background culling, the point cloud is clustered and selected as shown in Figure 11.

**Figure 12:**
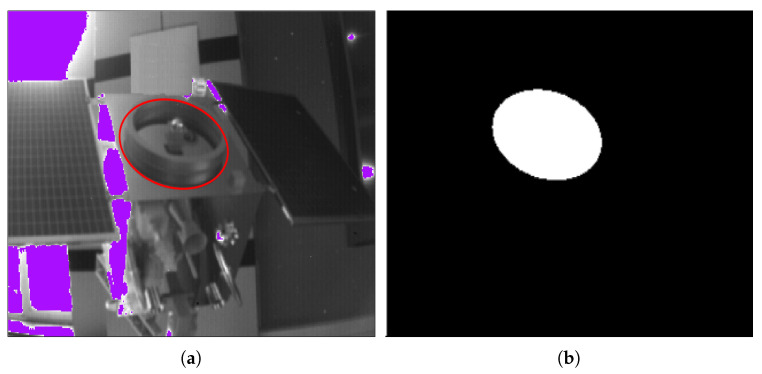
(**a**) The result of ellipse detection; (**b**) the region of interest.

**Figure 13:**
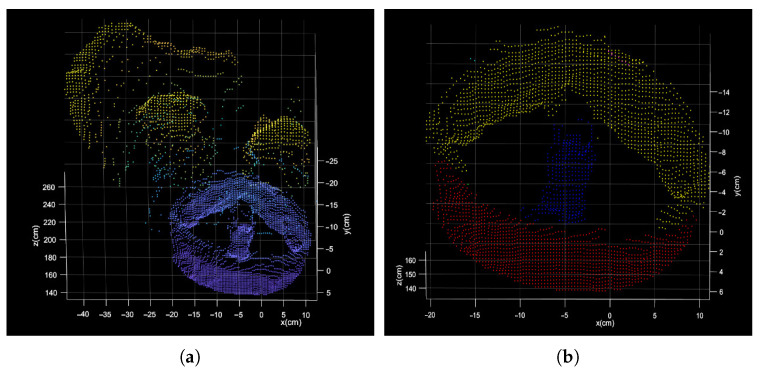
(**a**) The result of point cloud segmentation assisted by gray images; (**b**) the result after the clustering segmentation.

**Figure 14:**
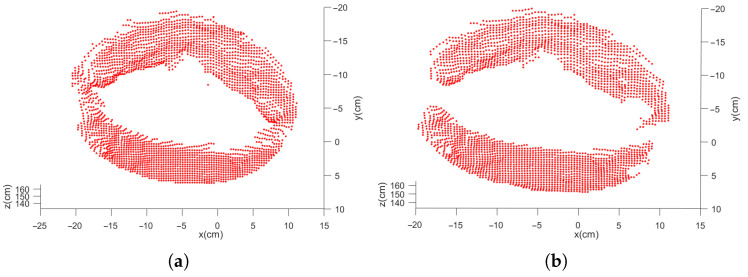
(**a**) The final results of the direct segmentation; (**b**) final results of the segmentation assisted by gray image.

The process of segmentation assisted by gray image is shown in Figure 12 and Figure 13. After ellipse detection, extract the region of interest and transform it to the local point cloud. The final circle feature can be obtained by Easy clustering and selecting. The result of the two methods is shown in Figure 14.

Table 1 shows the comparison of the two point cloud segmentation algorithms. With regard to the direct method, the data conversion takes most of the total time and a huge point number of point clouds is to be divided, which leads to a long segmentation time. Compared with the direct method, the gray image auxiliary method adds an ellipse-detection step and fuses the data conversion and circle feature extraction. In the gray image auxiliary method, the total time consumption is saved by 94.72% and the number of points in the point cloud to be divided is reduced by 93.53%.

The proposed method focuses the circle feature, which only occupies a part of the point cloud, thus the number of points to be processed is decreased. Since the total segmentation time is proportional to the number of points in the point cloud to be divided, the time efficiency is increased at a similar rate compared to the number improvement. It can be also found that the time improvement is higher than that of the number, which means that the algorithm has a good performance on the fast segmentation besides the data filtering (bilateral filters).

### 5.2. Relative Pose Accuracy and Robustness of Non-Ideal Circle Features of Point Clouds

In this section, we conduct a simulation test with the method of circle feature parameter fitting and pose calculation by geometric model to discuss the algorithm accuracy and the robustness against the noises. First, 100 groups of circle feature point cloud observation data are generated randomly and added by different levels of noises. After that, the algorithm is executed to solve the relative pose. Finally, the results are analyzed and calculated.

**Figure 15:**
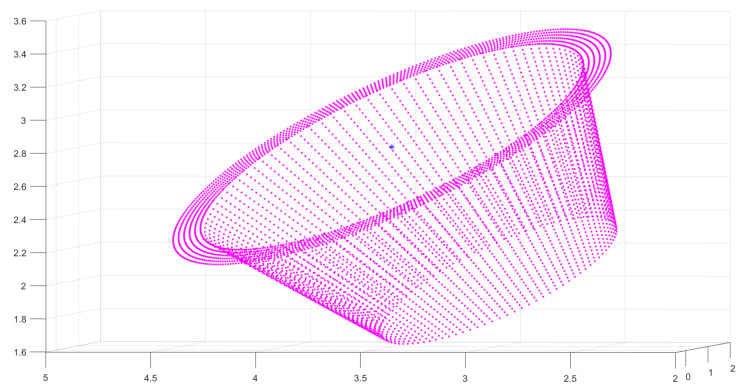
Standard model of circle feature.

In order to simulate the segmented circle feature observation point cloud, some steps are proceeded:

(1) Standard model settings: The standard model is a point cloud of the frustum of a cone with a resolution of 2 mm (as shown in Figure 15). The upper bottom’s radius is 12 cm, which is fixed on the mounting surface. On the standard plane, the lower bottom’s outer radius is 24 cm and the inner radius is 20 cm, which is the end face. The height of the model is 10 cm. Furthermore, the standard position of the circle feature model is fixed to the origin in CRF, i.e., the center of the end face coincides with the origin (0,0,0). The Euler angles are all 0 and the transformation matrix is the identity matrix I.
(2) Noise addition: Different levels of noises (0.01–1 magnitude of the resolution of point cloud standard model) are added to the standard model to verify the algorithm’s strong anti-interference ability.
(3) Model transformation: 100 groups of positions and Euler angles are generated randomly as nominal values. The final circle feature observation point cloud is generated by the noise-added standard model transformation according to the nominal values.

**Figure 16:**
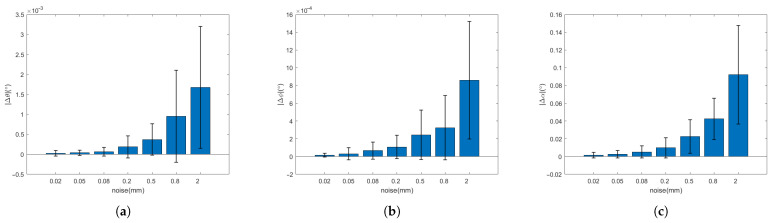
Errors of angles. (**a**) yaw angle; (**b**) pitch angle; (**c**) the included angle.

**Figure 17:**
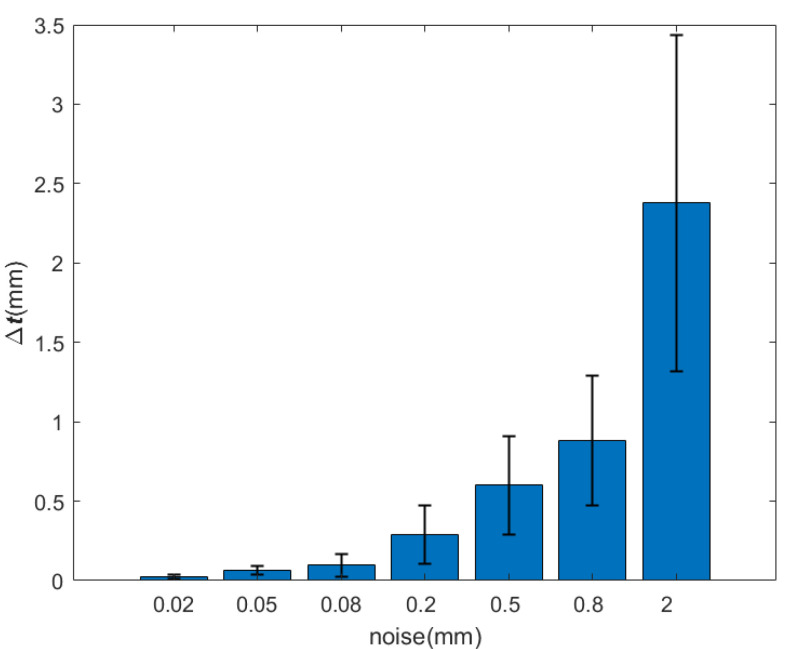
Standard model of circle feature.

The relative pose is obtained by the circle feature parameter fitting algorithm and the geometric model. The errors of the corresponding variables can be received by comparing the pose result with the nominal value. The final statistical results are as follows:

It can be seen from Table 2 and Table 3 and Figure 16 and Figure 17 that, when the observation model is added the noise of the order of 0.01–1 mm (the point cloud resolution is on the order of a millimeter), the errors of Euler angle and displacement increase with the increasing of the noise. However, the Euler angle always maintains high accuracy, whose total attitude angle error is within 0.1${}^{\circ }$ and the fluctuation range is within 0.1${}^{\circ }$. With respect to the displacement, when the noise is on the order of a millimeter, the error and fluctuation range is within several millimeters. With the noise under millimeter order, the accuracy can reach sub-millimeters or even higher. It can be seen that the relative pose solution method based on the circle feature point cloud has higher accuracy and better robustness under strong noises.

## 6. Conclusions

In this paper, we propose a relative pose determination strategy of uncooperative spacecraft in close-proximity operations. The natural circle feature acquired by the Time-of-Flight (ToF) camera is used to build the pose solution model based on specific coordinate frames. To improve the efficiency of the circle feature point cloud segmentation, gray images are used to assist the process of segmentation. These gray images are generated from the ToF camera together with depth images, which can be converted into the point cloud. In addition, the point cloud extraction algorithm solves the poor results from the bad ellipse detection results of the images. A novel circle feature fitting method is developed in order to cope with the non-ideal, or even variant shape of the circle feature in different on-orbit service scenarios.

The experiment showed that the proposed gray-image-assisted segmentation method dramatically decreased the computation time compared with the direct segmentation method. Numerical simulation results demonstrated that the accuracy of the attitude angle errors was kept within 0.1${}^{\circ }$ and the target displacement errors were at the point cloud resolution level when the maximum noise is at the level of point cloud resolution. In case the noise is lower than the resolution level, higher accuracy can be obtained with stronger robustness.

In the following research, the information of gray images from the ToF camera is expected to be deeply used. The method of fusing gray and depth image data will be considered for more robust and accurate pose estimation.

## Figures and Tables

**Table 1:** Comparison of point cloud segmentation algorithm results.

	Direct Method	Gray Image Auxiliary Method	Improvement (%)
Ellipse Detection Time (s)	—	0.047	—
Data Conversion Time (s)	26.625281	1.764461	—
Circle Segmentation Time (s)	7.687266		—
Total Time (s)	34.312547	1.811461	94.72
Divided Point Number	76,800	4970	93.53

**Table 2:** Statistics of error mean and variance of two-axis attitude.

Noise(mm)	Error phi (${}^{\circ }$)		Error Theta (${}^{\circ }$)		Error Angle (${}^{\circ }$)	
	Mean	Variance	Mean	Variance	Mean	Variance
0.02	1.5986 × $10^{-5}$	2.1604 × $10^{-5}$	2.2504 × $10^{-5}$	7.1102 × $10^{-5}$	0.0014	0.0031
0.05	3.0365 × $10^{-5}$	6.8873 × $10^{-5}$	3.8961 × $10^{-5}$	6.6372 × $10^{-5}$	0.0025	0.0042
0.08	6.6854 × $10^{-5}$	9.6205 × $10^{-5}$	6.6996 × $10^{-5}$	0.0001	0.0052	0.0067
0.2	0.0001	0.0001	0.0002	0.0003	0.0099	0.0114
0.5	0.0002	0.0003	0.0004	0.0004	0.0225	0.0189
0.8	0.0003	0.0004	0.0010	0.0012	0.0424	0.0233
2	0.0009	0.0007	0.0017	0.0015	0.0921	0.0555

**Table 3:** Displacement error.

Noise(mm)	Position(mm)	
	Mean	Variance
0.02	0.0250	0.0152
0.05	0.0620	0.0278
0.08	0.0977	0.0721
0.2	0.2909	0.1844
0.5	0.6009	0.3109
0.8	0.8829	0.4063
2	2.3774	1.0606
